# BDNF Val66Met polymorphism, energy intake and BMI: a follow-up study in schoolchildren at risk of eating disorders

**DOI:** 10.1186/1471-2458-10-363

**Published:** 2010-06-23

**Authors:** Victoria Arija, Marta Ferrer-Barcala, Nuria Aranda, Josepa Canals

**Affiliations:** 1 IISPV, Unitat de Medicina Preventiva i Salut Pública., Universitat Rovira i Virgili, Spain; 2 CRAMC, Departament de Psicologia. Universitat Rovira i Virgili, Spain

## Abstract

**Background:**

Eating disorders (ED) have a multifactorial aetiology in which genetics play an important role. Several studies have found an association between the Val66Met (G196A) polymorphism of the Brain-Derived Neurotrophic Factor (BDNF) and Eating disorders.

The aim of this study was to determine the association of the Val66Met (G196A) polymorphism of the BDNF gene and its effect on eating disorders (ED), energy intake and BMI in schoolchildren.

**Methods:**

Two-year cohort study (preadolescence to adolescence). From an initial sample of 1336 Caucasian children (mean age = 11.37 years), a group at risk of ED (n = 141) and a control group (n = 117) were selected using the Children's Eating Attitudes Test. Two years later, they were re-classified into an at-risk group (n = 41) and a control group (n = 159) using the Eating Attitudes Test. The diagnosis of the individuals at risk of ED was confirmed by means of the Diagnostic Interview for Children and Adolescents. BMI, energy intake and the Val66Met (G196A) polymorphism of the BDNF gene were analysed in the at-risk and control groups.

**Results:**

The frequency of genotypes of the Val66Met (G196A) polymorphism of the BDNF gene is 28.6% (95% CI: 22.4-34.9) in the heterozygous form (Val/Met) and 5% (95% CI: 2.4-9) in the homozygous form (Met/Met). We detected no association between Val66Met genotypes and the severity of ED. Over time, the carriers of the Met66 allele with a persistent risk of ED significantly restricted energy intake (507 Kcal/day; p = 0.033).

**Conclusion:**

We have not found an association between Val66Met (G196A) polymorphism of the BDNF and ED in schoolchildren from general population. The relationship found between this polymorphism and energy intake restriction in adolescents with a persistent risk of ED should be replicated in a larger sample.

## Background

Eating disorders (ED) have a multifactorial aetiology and negatively affect both the psychosocial and developmental levels. A high proportion of adolescents--between 20% and 25%--have inappropriate attitudes towards weight control [1-3]. The continuity hypothesis of ED supported by Vanderheyden and Boland [4] suggests that risky attitudes could be responsible for ED [5,6] and may be related to the same biological and personality characteristics that are present in full-blown syndromes.

Many studies have investigated the role of genetics in ED. Brain-derived neurotrophic factor (BDNF) is a protein belonging to the neurotrophin family that is involved in the proliferation, differentiation and survival of neurons in the nervous system [7]. In the past few years, it has generated considerable interest because of its distribution in the key brain regions that are involved in behaviour, energy homeostasis and weight control. Furthermore, BDNF provides trophic support to noradrenergic, dopaminergic and serotonergic neurons, among others [8], whose neurotransmitters are altered in many mental illnesses. Some researchers have hypothesised that neurotrophins are involved in mental illnesses because of an alteration in synaptic plasticity [9,10], in which BDNF plays a fundamental role. This synaptic alteration could be due to the Val66Met (G196A) polymorphism of the BDNF gene (MIM 113505), which alters how the secretion of mature BDNF is regulated [11,12]. This polymorphism (Single Nucleotide Polymorphism database (dbSNP) rs6265) is located on chromosome 11p13-11p14 [13], which encodes the peptide precursor (proBDNF) of BDNF.

Restricting anorexia nervosa (AN) and minimum body mass index (BMI) has been associated with the aforementioned Val66Met (G196A) polymorphism of the BDNF gene, and it has been suggested that the A allele (Met) may affect susceptibility to ED [14,15]. In a broad population from six samples in five European countries, consisting mainly of women aged 20-30, the association of the polymorphism was extended to all kinds of ED, including restricting AN (ANR), binge-eating AN (ANB) and bulimia nervosa (BN) [14]. In Japan, Koizumi et al [16] observed significant differences between the genotypes of the Val66Met (G196A) polymorphism in female patients with ED (EDNOS, AN and BN) and those of control participants. When observed separately, however, significant differences were found only in the distribution of genotypes in ANR and purging BN. A significant association was also found between a higher score on the Bulimic Investigatory Test, Edinburgh (BITE) and the AA genotype (Met/Met) in patients with BN and binge ED [17].

A recent meta-analysis study [18] suggested that Caucasian individuals aged 20-26 with Val/Met genotypes have a 36% higher risk of developing ED than those with the Val/Val genotype. However, some studies have found there to be no association in patients with ED [17,19,20].

BDNF is also known to play a key role in eating behaviour and weight regulation [21]. Both BDNF and its receptor NTRK2 are found in hypothalamic areas that are critical to feeding and energy regulation [22]. Studies carried out on animals suggest that hyperphagia affects not only the relationship between the level of BDNF and BMI [23], but also the regulation of food intake by acting as an anorexigenic factor [24,25].

Attempts have been made to establish an association between polymorphism and BMI in the general adult population of both sexes, but the results are contradictory. Compared with the Val/Met and Val/Val genotypes, the association between the Met/Met genotype and low BMI is much greater [26]. Nevertheless, contrary to the results of a recent study [27], we observed that patients with BN carrying the Met66 allele of the BDNF gene and the 7R allele of the dopamine-4 receptor gene (DRD4) have a higher BMI. Mutations in the gene that encodes the BDNF or its receptor (TrKB) may explain certain kinds of obesity or forms of ED in humans [25].

In light of the lack of research on the frequency of BDNF gene alteration in the adolescent population, the contradictory results reported to date with regard to the association between BDNF gene alteration and ED, and the main consequences of this type of alteration on energy intake and BMI, we decided to undertake a longitudinal study to clarify these aspects. The objectives of our study were: a) to determine the relation between the frequency of the Val66Met (G196A) polymorphism of the BDNF gene and the severity of risk of ED, b) to establish the association between genetic alteration and risk of ED, and c) to study the effects of this genetic alteration on BMI and energy intake according to the evolution of the risk of ED.

## Methods

### Design

Two-year cohort study.

### Participants

**Figure 1:**
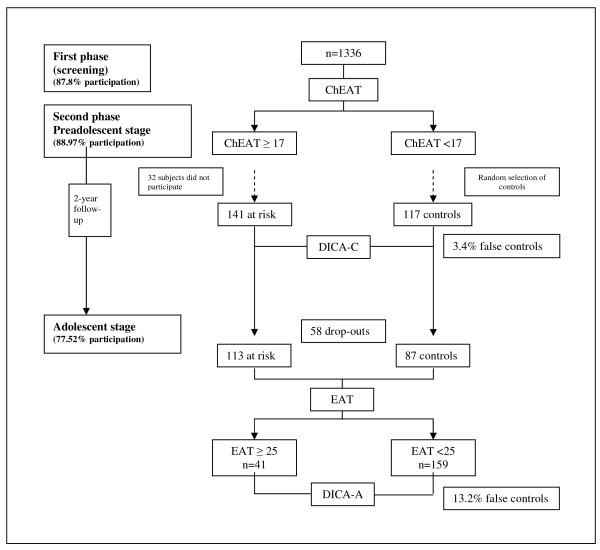
**Description of the sample**. ChEAT: Children's Eating Attitudes Test; DICA-C: Diagnostic Interview for Children and Adolescents, children's version; EAT: Eating Attitudes Test; DICA-A: Diagnostic Interview for Children and Adolescents, adolescents' version. Data published by Sancho et al [28].

We studied 258 Spanish schoolchildren of Caucasian origin of both sexes aged 10-13 (mean = 11.37, SD = 0.62) taken from an initial sample of 1,336 schoolchildren (649 boys and 687 girls). The participants were taken from 17 randomly selected primary schools so that the group was representative of the social and cultural backgrounds of various places (cities and suburbs) within the province of Tarragona (Spain). This sample was studied in two phases. In the first phase of the study, an ED screening test (Children's Eating Attitudes Test, or ChEAT) was administered to the entire sample in order to select at-risk schoolchildren. A total of 141 were included in the at-risk group and 117 were included in the control group; the ages of the children ranged from 11 to 13 (mean = 11.31, SD = 0.59). In the preadolescent stage of the second phase, controls were randomly selected from those whose score on the ChEAT was at or below the cut-off and whose characteristics of age, gender and type of school were similar. Two years later, in the adolescent stage, the individuals in the sample from the second phase, now aged 13-15 (mean = 13.79, SD = 0.72) were assessed once again. Of these individuals, a total of 113 at-risk individuals and 87 controls were identified. These subjects were given the Eating Attitudes Test (EAT), and 41 were found to be at risk and 159 were selected as controls. Figure 1 gives a description of the sample. For further information, see Canals F, et al. (in press) and Sancho C et al [28].

The reasons for non-participation were lack of parental consent, absence from school or refusal by the potential subject. The percentages of participation were 87.8% in the screening phase and 88.97% in the preadolescent stage. Of these, 77.52% were monitored in the adolescent stage.

### Procedure

Before starting the study, we obtained permission from the Catalan Government and from the schools' headmasters. Informed consent was also obtained from the children's parents in both phases of the study. The study was approved by the Ethics Committee for Clinical Research at the Sant Joan University Hospital in Reus (Spain).

In the first stage of the study, the preadolescent participants in the initial sample took the adapted ChEAT [29] in the classroom while the researchers (a psychologist and a dietician) were present. We took a sample of the participants who scored at least 17 (the ChEAT cut-off score) and a control group of participants who scored below the cut-off by selecting two out of every three cases. The at-risk participants and controls were weighed and measured, and their BMIs calculated. Their energy intake was assessed by their parents with a three-day food record for two non-consecutive weekdays and one weekend day (and confirmed by the children with a photograph album of dishes and portions). The ED section of the Diagnostic Interview for Children and Adolescents (children's version, DICA-C) was used to make individual assessments.

Two years later, we phoned the families that had participated in the preadolescent stage to inform them that the adolescent stage of the study was to be held. Individuals who agreed to participate again were assessed using the EAT [30] and were weighed and measured so that their BMIs could be calculated. In this stage we used the EAT because it is a reliable test for adolescents but not for children. Participants were also assessed individually using the ED section of the Diagnostic Interview for Children and Adolescents (adolescents' version, DICA-A). Energy intake was assessed by means of a 24-hour recall interview for three non-consecutive days, including one which was not a weekday. We collected samples of oral epithelium so that we could subsequently analyze the Val66Met polymorphism of the BDNF gene. We used double-blind evaluation methods in both phases.

We classified the participants into four groups defined by the evolution of the risk of ED according to the results of the ChEAT and the EAT: A) no risk in either stage (No→No); B) risk in the first stage but not in the second stage (Yes→No); C) no risk in the first stage but risk in the second stage (No→Yes); and D) risk in both the first and second stages (Yes→Yes). These groups were checked using the DICA-C and the DICA-A in order to eliminate false controls, which accounted for 3.4% in the preadolescent stage and 13.2% in the adolescent stage. We classified participants into four groups in order to study the evolution of ED according to the DICA-C (preadolescent stage) and the DICA-A (adolescent stage). We considered individuals with subclinical syndromes, EDNOS and full syndromes as part of the ED diagnostic group [28].

We used DSM-IV criteria to define the cases of EDNOS and subclinical syndromes on the basis of information collected with the DICA-C and the DICA-A. We defined participants with subclinical anorexia as those who fulfil the psychopathological criteria of ED based on fear of gaining weight (B criteria for AN) or self-esteem that is completely based on body weight and shape (C criteria for AN), but who do not satisfy clinical criteria such as amenorrhea (D criteria for AN) or weight loss (A criteria for AN). For subclinical bulimia, we did not consider the frequency of binge eating (C criteria for BN) or the loss of control over eating during the episode (A-2 criteria for BN).

### Instruments

### Assessment of risk of ED and diagnoses

#### Children's Eating Attitudes Test (ChEAT) [31]

This 26-item scale is designed to assess maladaptive or problematic eating attitudes and behaviours among children. In this study, we used a 20-item experimental adapted Spanish version (reliability α = 0.73) to detect possible risk of ED and used a cut-off score of 17 [29].

#### Eating Attitudes Test (EAT) [32]

This 40-item self-report has six possible answers and is designed to assess ED behaviours. It has good psychometric characteristics of reliability and validity. In this study, we administered the Spanish validated version of the EAT (internal consistency α = 0.93) [30]. We used a cut-off score of 25 or more because it has been shown to provide better data on the sensitivity (87.5%) and specificity (93.9%) of young populations [33].

#### Diagnostic Interview for Children and Adolescents (DICA)

The Spanish computerised adaptations [34] of the DICA-R (Diagnostic Interview for Children and Adolescents, Revised) and the DICA-IV [35,36] were administered to diagnose ED. The DICA is a semi structured interview for children (DICA-C) and adolescents (DICA-A) that follows DSM-IV diagnostic criteria [37]. Although the DICA covers several psychiatric diagnostic areas, in this study we only administered the ED diagnostic area, which covers AN and BN. The Kappa statistics for the test-retest reliability of the Spanish version for ED were between 0.74 and 1.0 [34]. We made diagnoses on the basis of information obtained with the DICA-C in the first (preadolescent) stage and with the DICA-A in the second (adolescent) stage.

### Anthropometric measurements and assessment of energy intake

Food consumption was assessed quantitatively over the course of three non-consecutive days, one of which was not a weekday. Because adolescents are more aware than preadolescents of the food they have consumed and of family consumption habits, we used a different method for assessing food consumption that was better adapted to their characteristics and gave better results. In the preadolescent stage, we used a food record [38], whereas in the adolescent stage we used a 24-hour recall method [39,40].

#### Food record

The parents or guardians of preadolescents recorded the quantity of food and drink consumed by their children. They expressed these quantities using simple measurements and gave information about how the food was prepared, what ingredients were used and at what time their children ate. The data were checked in a meeting with the parents and subsequently with the child. Particular care was taken to determine food intake outside of the home. This method was carried out by trained nutritionists.

#### 24-hour recall

This method involved interviewing the adolescents. An extensive collection of photographs with a variety of portion sizes was used to provide a better assessment of the food intake. The interviews were carried out by trained nutritionists.

In both phases, energy intake was calculated using the French REGAL food composition table [41], and the Spanish food composition table [42] was used for foods specific to Spain.

#### Anthropometric assessment

The BMI was calculated by dividing the participants' weight in kilograms by their size in square metres (kg/m^2^). Height was measured using an ordinary tape measure, with the participants standing upright and wearing no shoes or headwear. We checked that all participants remained still, with their heels in line with the longitudinal axes of both feet. Weight was calculated using a digital scale (Tanita 305) equipped with an impedance analyzer. The participants were weighed in the morning, before their morning break at school. They had to wear as little clothing as possible and go barefoot in order to prevent interference with the bio impedance.

### DNA extraction and genotyping

We collected samples of oral epithelium using sterilised swabs after the children had not eaten or drunk anything for at least 30 minutes. The samples were kept at room temperature on specimen collection cards (S&S 903^®^, Schleicher & Schuell BioScience Inc., Keene, NH, USA) and stored in individual bags with a packet of desiccant.

The genomic DNA was extracted using isolation reagents from a Puregene kit (Gentra Systems, USA). In samples in which little DNA was extracted or the DNA was weak, the entire genomic DNA was amplified using a GenomiPhi kit (GE Healthcare, USA). The DNA was extracted and amplified at the BioBank of the Institute for Health Sciences Research (IRCIS) at Sant Joan University Hospital in Reus (Spain). In one case, the DNA extraction was not satisfactory.

The G196A variant of the BDNF gene was genotyped using the polymerase chain reaction-restriction fragment length polymorphism (PCR-RFLP) technique. The SNP in the BDNF gene was categorised for each DNA sample using a PCR with a total volume of 10 μl, consisting of 20 ng of DNA, H_2_O, 1× gold buffer (GeneAmp^® ^gold buffer, Applied Bio systems), 1.5 mM MgCl_2_, 0.2 mM dNTPs, 0.5 μM BDNF-F (5'-AGGTGAGAAGAGTGATGACC-3'), 0.5 μM BDNF-R (5'-CTGGACGTGTACAAGTCTGC-3') and 0.5 U *Taq *DNA polymerase (AmpliTaq^® ^gold DNA polymerase, Applied Bio systems). The initial conditions of the PCR were 94°C for 5 min followed by 32 cycles of 30 s at 94°C, 30 s at 58°C and 30 s at 72°C, and finally 7 min at 74°C, until a 292 bp product was obtained. Digestion was then carried out using the *NlaIII *restriction enzyme. For an enzyme volume of 2 μl, it contained 1× NE buffer 4, 1 U *NlaIII *and H_2_O. Digestion was carried out at 37°C for 1 h 15 min. Digestion with *NlaIII *produced four fragments with 160, 59, 58 and 15 bp, respectively, in the presence of the Met66 allele, and five fragments with 83, 77, 59, 58 and 15 bp, respectively, in the presence of the Val66 allele. Finally, electrophoresis was applied to a polyacrylamide gel at 12% with a TBE buffer.

The genotyping was undertaken by the IRCIS UniLab at the Sant Joan University Hospital in Reus (Spain).

### Statistical analysis

The categorical variables have been presented as percentages and their corresponding confidence interval, and the quantitative variables (BMI and energy intake) as the median and the interquartile range because of its skewed distribution.

The genotypes were stratified by the severity of ED risk. The genotype distribution at different severities of ED risk was tested for Hardy-Weinberg equilibrium by chi-square test or Fisher's exact test when necessary. Mann-Whitney U test, with Bonferroni correction to solve the multiple testing problems, were used to compare BMI and Energy intake in the different groups. Wilcoxon test were used to observe the relationship between genotype and repeated measures of BMI and energy intake in the same subjects. All data were analyzed using the SPSS package for Windows (version 17.0). The level of statistical significance was set at p < 0.05.

## Results

The frequency of the genotypes of the Val66Met (G196A) polymorphism of the BDNF gene does not depend on the severity of ED risk in girls (*χ*^2 ^= 2.96; d.f. = 4; p = 0.562) or in boys (*χ*^2 ^= 6.25; d.f. = 4; p = 0.181) (Table 1).

**Table 1:** Frequency of the Val66Met (G196A) genotypes according to the risk of ED in preadolescence

ED severity		**Val/Val**^**a**^		**Val/Met**^**b**^		**Met/Met**^**c**^		p-value	p-value a vs b+c
		n % (95% CI)		n % (95% CI)		n % (95% CI)			
	Risk	32	76.2 (60.6-87.9)	9	21.4 (10.3-36.8)	1	2.4 (0.0-12.6)		
Male	Diagnosis	7	43.8 (19.8-70.1)	7	43.8 (19.8-70.1)	2	12.5 (1.6-38.3)	0.181	0.099
	Control	22	64.7 (46.5-80.3)	9	2 6.5 (12.9-44.4)	3	8.8 (1.9-23.7)		

	Risk	28	68.3 (51.9-81.9)	12	29.3 (16.1-45.5)	1	2.4 (0.1-12.9)		
Female	Diagnosis	17	73.9 (51.6-89.8)	6	26.1 (10.2-48.4)	0	-	0.562	0.527
	Control	25	61.0 (44.5-75.8)	13	31.7 (18.1-48.1)	3	7.3 (1.5-19.9)		

	Risk	60	72.3 (61.4-81.5)	21	25.3 (16.4-36.0)	2	2.4 (0.3-8.4)		
All	Diagnosis	24	61.5 (44.6-76.6)	13	33.3 (19.1-50.2)	2	5.1 (0.6-17.3)	0.433	0.199
	Control	47	62.7 (50.7-73.6)	22	29.3 (19.4-41.0)	6	8.0 (3.0-16.6)		

CI: Confidence interval, Diagnosis: any diagnosis

Table 2 shows the distribution of the genotypes of the polymorphism according to the evolution of risk and diagnosis of ED in both sexes. No statistically significant differences were found between the groups for the evolution of risk (*χ*^2 ^= 6.44; d.f. = 6; p = 0.381) or of ED diagnosis (*χ*^2 ^= 3.097; d.f. = 6; p = 0.797).

**Table 2:** Distribution of the Val66Met (G196A) genotypes according to the longitudinal evolution of the risk and diagnosis of ED

Evolution of risk						Evolution of diagnosis				
	**Val/Val**^**a**^	**Val/Met**^**b**^	**Met/Met**^**c**^			**Val/Val**^**a**^	**Val/Met**^**b**^	**Met/Met**^**c**^		
	n % (95% CI)	n % (95% CI)	n % (95% CI)	p-value	p-value a vs b+c	n % (95% CI)	n % (95% CI)	n % (95% CI)	p-value	p-value a vs b+c
**No→No**	42 66.7 (53.7-78.0)	16 25.4 (15.3-37.9)	5 7.9 (2.6-17.6)			89 68.5 (60.5-76.4)	35 26.9 (19.3-34.5)	6 4.6 (1.7-9.8)		
**Yes→No**	49 66.2 (54.3-76.8)	23 31.1 (20.8-42.9)	2 2.7 (0.3-9.4)	0.381	0.343	9 50.0 (26.0-74.0)	8 44.4 (21.5-69.2)	1 5.6 (0.1-27.3)	0.797	0.298
**No→Yes**	5 41.7 (15.2-72.3)	6 50.0 (21.1-78.9)	1 8.3 (0.2-38.5)			18 64.3 (44.1-81.4)	8 28.6 (13.2-48.7)	2 7.1 (0.9-23.5)		
**Yes→Yes**	35 72.9 (58.2-84.7)	11 22.9 (12.0-37.3)	2 4.2 (0.5-14.3)			15 71.4 (47.8-88.7)	5 23.8 (8.2-47.2)	1 4.8 (0.1-23.8)		

CI: confidence interval; No→No: no risk/diagnosis in either preadolescence or adolescence; Yes→No: risk/diagnosis in preadolescence but no risk/diagnosis in adolescence; No→Yes: no risk/diagnosis in preadolescence but risk/diagnosis in adolescence; Yes→Yes: risk/diagnosis in both preadolescence and adolescence.

The distribution of genotypes according to the severity of ED did not differ significantly from that expected from the Hardy-Weinberg equilibrium in the control group (*χ*^2 ^= 0.06; d.f. = 2; p = 0.970) (*χ*^2 ^= 5.37^-3^; d.f. = 2; p = 0.977), the risk group (*χ*^2 ^= 3.48^-3^; d.f. = 2; p = 0.998) (*χ*^2 ^= 0.04; d.f = 2; p = 0.981) or the ED diagnosis group (*χ*^2 ^= 0.04; d.f. = 2; p = 0.980) (*χ*^2 ^= 0.45; d.f. = 2; p = 0.798) in girls or boys, respectively. Nor was the genotype distribution significantly different in the groups classified according to evolution of risk and evolution of ED.

Table 3 shows the energy intake and BMI for both sexes in the two phases of the study and compares the control group, risk group and diagnostic group. The BMI is higher where there is a higher level of severity of ED. There are significant differences between the control group and the diagnostic group in preadolescent and adolescent females (p < 0.001 and 0.012 respectively) and in preadolescent and adolescent males (p < 0.001 and 0.012 respectively). In the at-risk and diagnosis groups, however, energy intake is lower, especially in adolescent girls, and there is a statistically significant difference between the control group and the diagnostic group (p = 0.003).

**Table 3:** Energy intake and BMI according to the severity of the ED in preadolescence and in adolescence

		Females					Males				
		Preadolescence		Adolescence		p-value	Preadolescence		Adolescence		p-value
		n	Median(IQR)	n	Median(IQR)		n	Median(IQR)	n	Median(IQR)	
	Control^a^	37	2305(678)	50	2266(804)	0.216	30	2601(650)	68	2610(1194)	0.347
	Risk^b^	38	2193(599)	9	1871(780)	0.079	36	2554(734)	2	1913(-)	0.191
**Energy intake (Kcal/day)**	Diagnosis^c^	20	2070(617)	36	1621(745)	0.058	10	2468(963)	6	2267(946)	0.588
	p-value^a-b^		0.203		0.15			0.807		0.225	
	adjusted p-value^a-b^		0.406		0.3			1		0.45	
	p-value^a-c^		0.032		0.003			0.656		0.137	
	adjusted p-value^a-c^		0.064		0.006			1		0.274	

**BMI (kg/m^2^)**	Control^a^	36	18.6(4.2)	50	20.5(5)	0.001	30	14.3(3.9)	68	20.2(5)	0.002
	Risk^b^	38	19.9(5.3)	9	24.9(11)	0.027	35	19.5(4.5)	2	23.9(-)	0.077
	Diagnosis^c^	20	23.7(5.3)	36	23.5(6)	0.758	10	22.7(1.9)	6	24.5(3)	0.039
	p-value^a-b^		0.016		0.007			0.276		0.174	
	adjusted p-value^a-b^		0.032		0.14			0.552		0.348	
	p-value^a-c^		<0.001		0.006			<0.001		0.006	
	adjusted p-value^a-c^		<0.001		0.012			<0.001		0.012	

IQR: Interquartil range; adj.:adjusted; Diagnosis: any diagnosis; The adjusted p-value were corrected with the Bonferroni test (post-hoc comparisons between the 2 groups).

**Figure 2:**
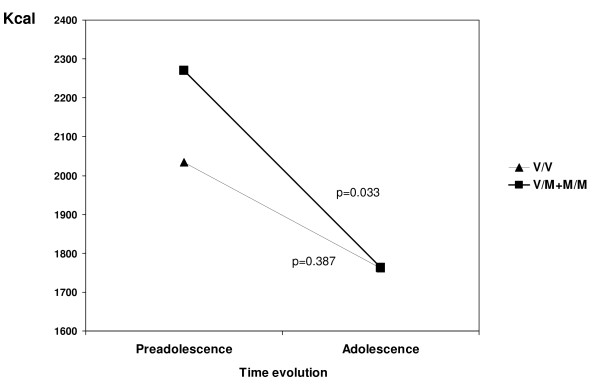
**Energy intake in persistent risk of ED group according to the Val66Met (G196A) genotypes**. V/V: Val/Val; V/M: Val/Met; M/M: Met/Met.

Table 4 shows the energy intake and BMI for the groups based on evolution of risk of ED and the gene polymorphism in both sexes. Significant differences were found between the evolution in the BMIs of carriers of the Met allele (Val/Met, Met/Met) and of carriers of the wild allele (Val/Val) in all the groups. Significant differences in energy intake were found in the carriers of the Met allele (p = 0.033) only in the Yes→Yes risk group (Figure 2).

**Table 4:** BMI and energy intake in the groups of evolution of risk of ED according to the Val66Met (G196A) genotypes

			BMI (kg/m^2^)			Energy intake (Kcal/day)		
		**n**	**Preadolescence** **Median(IQR)**	**Adolescence** **Median(IQR)**	**p-value**	**Preadolescence** **Median(IQR)**	**Adolescence** **Median(IQR)**	**p-value**

**No→No**	V/V	36	18.4(4.2)	20.5(5)	<0.001	2357(717)	2659(1340)	0.102
	V/M + M/M	21	18.4(4.3)	21(6)	<0.001	2418(682)	2448(871)	0.274

**Yes→No**	V/V	41	19.4(5.4)	21.3(6)	<0.001	2300(689)	2311(1102)	0.750
	V/M + M/M	20	20.7(5.4)	20.9(5)	0.02	2623(761)	2477(861)	0.277

**No→Yes**	V/V	4	18.4(3)	22.1(2)	0.043	2120(1522)	2127(541)	0.686
	V/M + M/M	6	20.4(4.7)	22.6(6)	0.028	2431(870)	1794(650)	0.075

**Yes→Yes**	V/V	31	22.9(5.3)	24.7(8)	0.001	2034(670)	1762(1137)	0.387
	V/M + M/M	11	22.9(4.6)	24.6(5)	0.016	2270(771)	1763(490)	0.033

IQR: Interqualtin range; No→No: no risk of ED in either preadolescence or adolescence; Yes→No: risk in preadolescence but no risk in adolescence;

No→Yes: no risk in preadolescence but risk in adolescence; Yes→Yes: risk in both preadolescence and adolescence; V/V: Val/Val; V/M: Val/Met;

M/M: Met/Met. No significant differences between V/V and V/M + M/M genotypes in either group

In the groups based on the evolution of ED diagnosis, no significant differences were observed in either energy intake or BMI (data not shown in the table).

## Discussion

In this study, we analysed a genetic component, the Val66Met (G196A) polymorphism of the BDNF gene, in a sample of schoolchildren susceptible to developing some kind of ED. The longitudinal design allowed us to observe the evolution of risk of ED and how it is associated with the genotypes of the polymorphism. The frequency of the polymorphism in our sample contributes to the intrapopulation differences in Val66Met in the Caucasian population. According to Shimizu et al., the distribution of genotypes varies according to population: participants of Asian origin carry a higher percentage of the Met66 allele than participants of Caucasian origin [43].

Our results do not reveal any association between the Val66Met (G196A) polymorphism of the BDNF gene and the different levels of severity of risk of ED, the evolution of risk, or ED diagnosis. These results support those obtained for clinical populations of participants diagnosed with BN [17,20] and AN [19,20]. However, they contradict the positive results obtained by Ribasés et al. [14,15,44] and Koizumi et al. [16] for all kinds of ED.

According to various studies, the prevalence of heterozygote (Val/Met) in AN is about 35.8%, with values ranging from 24.4% to 45.9%, while the prevalence of homozygote (Met/Met) is 3.6% (2.3-4.9%) [20,44]. In BN, the average prevalence of the heterozygote is 34.8% (26.9-42.4%) and the average prevalence of the homozygote is 5.2% (2.2-10.3%) [17,19,20]. In the control participants who took part in the above studies, the average prevalence of the heterozygote was 31.9% (28.4-34.4%) and the average prevalence of the homozygote was 3% (1.0-4.9%) [17,19,20]. In our study, we did not observe higher percentages of the Met allele at higher severities of risk of ED. Although there were no significant differences, a higher percentage of the Met allele was found among those schoolchildren who evolved from 'no risk' to 'at risk', which suggests susceptibility to ED [14,15,18,44]. Therefore, the association found between individuals carrying the Met allele who continued to be at risk of ED and their high restriction of energy intake (546 kcal/day) was important. The method used to measure intake has been validated internationally by epidemiologic studies [38,39,45].

As in other studies on ED [17,19,20], we did not find a relationship between Val66Met and BMI. Nevertheless, according to the results in table 3, the girls at risk of ED or with ED in preadolescence and adolescence were overweight and were dieting in adolescence. This may lead to lower BMI in the long term. However, in our sample [46], the most frequent diagnoses were not full-blown syndromes and there was no AN diagnosis. For this reason, and because of the early age of the participants, we observed no reduction in the BMI. However, as can be seen in table 4, only the Met allele carriers within the Yes-Yes risk group restricted their energy intake. Although this low intake may be due to under reporting, by using the Goldberg technique we found that these girls may really be restricting their intake [45]. Genetic factors, then, may interact with such variables as BMI, dieting, body dissatisfaction, and socio-cultural and emotional factors to increase the risk of ED [47]. In contrast, an association has been found between the Met allele and low BMI in both the adult clinical ED population [15], and more specifically in the ANR population [14], and the healthy adult population, in which the Met/Met genotype is associated with low BMI [26]. This leads us to hypothesise that the lack of a significant association in our study may be due to such factors as the age of the participants, the small sample size of each group based on the evolution and severity of ED, and the fact that our cases are less severe than those in the general population. The sex of the participants was also an important factor, since most clinical cases are women, whereas our study assessed both sexes. The reason why the polymorphism did not act in the age groups studied, then, may be that the participants had not reached neurodevelopmental maturity. We must also take into account that there were interactions between the Val66Met and other, unstudied genetic factors [48]. Kaplan et al. (2008) observed that interaction between the Met66 allele of the BDNF gene and the 7R allele of the dopamine-4 receptor gene (DRD4) is associated with a higher BMI in patients with BN. However, when they analysed Val66Met alone, they found that it had no effect on BMI. They hypothesized that the interaction of hypofunctional alleles increases intake, because of higher levels of circulating dopamine or low levels of BDNF, which results in a high BMI. Our results, however, did not show a higher energy intake in participants carrying the Met66 allele who were still at risk of ED in the follow-up and had a high BMI. This suggests that it is the BMI that triggers the onset of ED because it affects energy intake, particularly in carriers of the Val66Met polymorphism.

Although we observed no direct effect of the Val66Met polymorphism on those schoolchildren with some kind of ED, some previous studies [49,50] have established an indirect relationship between Val66Met and ED through personality traits. Sen et al. [50] associated the Val/Val genotype with neuroticism in Caucasians, specifically with the traits of anxiety, depression, self-consciousness, vulnerability and feelings. In Japanese women, the Met/Met genotype was associated with higher scores on the 'reward dependence' trait in the Temperament and Character Inventory (TCI) and on the 'extraversion' trait in the NEO Personality Inventory [49].

## Conclusions

A high percentage of the general population studied was shown to have the Val66Met (G196A) polymorphism of the BDNF gene (33.7%; 95% CI: 27.1-40.2). However, no direct relationship was observed between the presence of the polymorphism and risk of ED. Nevertheless, in one small group (adolescents with a persistent risk of ED for at least two years), the presence of the polymorphism leads to a greater restriction of energy intake (507 Kcal/day, p = 0.033) than that found in the general population. No direct relationship was found between BMI and the polymorphism. Nevertheless, further studies with larger samples and long-term monitoring are required if the evolution of ED is to be better understood.

## Competing interests

The authors declare that they have no competing interests.

## Authors' contributions

The study's chief researchers VA and JC were responsible for identifying the research question, designing the study, obtaining ethics approval, acquiring funding, analysing the data, writing the manuscript and overseeing the study. MF contributed to developing the precise content of the study interventions and resources, recruited participants, implemented the study and wrote the paper. MF and NA reviewed the literature and analyzed the data. All authors were responsible for drafting the manuscript, and read and approved the final version.

## Pre-publication history

The pre-publication history for this paper can be accessed here:

http://www.biomedcentral.com/1471-2458/10/363/prepub
